# Functional Outcomes and Quality of Life after Cochlear Implantation in Patients with Long-Term Deafness

**DOI:** 10.3390/jcm11175156

**Published:** 2022-08-31

**Authors:** Attila Ovari, Lisa Hühnlein, David Nguyen-Dalinger, Daniel Fabian Strüder, Christoph Külkens, Oliver Niclaus, Jens Eduard Meyer

**Affiliations:** 1 Department of Oto-Rhino-Laryngology, Head and Neck Surgery, Plastic Surgery, Asklepios Klinik St. Georg, 20099 Hamburg, Germany; 2 Asklepios Medical School, Semmelweis University, 20099 Hamburg, Germany; 3 Hanseatisches Cochlea Implantat Zentrum (HCIZ), 22417 Hamburg, Germany; 4 Department of Oto-Rhino-Laryngology, Head and Neck Surgery, Facial Plastic Surgery, Asklepios Klinik Nord-Heidberg, 22417 Hamburg, Germany; 5 Department of Oto-Rhino-Laryngology, Head and Neck Surgery “Otto Koerner”, University Medical Center, 18057 Rostock, Germany

**Keywords:** cochlear implants, long-term deafness, mid-scala electrode, Freiburger speech intelligibility test for monosyllables, Nijmegen Cochlear Implant Questionnaire, quality of life

## Abstract

Background: Hearing-related quality of life (QoL) after cochlear implantation (CI) is as important as audiological performance. We evaluated the functional results and QoL after CI in a heterogeneous patient cohort with emphasis on patients with long-term deafness (>10 years). Methods: Twenty-eight patients (*n* = 32 implanted ears, within *n* = 12 long-term deaf ears) implanted with a mid-scala electrode array were included in this retrospective mono-centric cohort study. Speech intelligibility for monosyllables (SIM), speech reception thresholds (SRT_50_) and QoL with Nijmegen Cochlear Implant Questionnaire (NCIQ) were registered. Correlation of SIM and QoL was analyzed. Results: SIM and SRT_50_ improved significantly 12 months postoperatively up to 54.8 ± 29.1% and 49.3 ± 9.6 dB SPL, respectively. SIM progressively improved up to 1 year, but some early-deafened, late implanted patients developed speech understanding several years after implantation. The global and all subdomain QoL scores increased significantly up to 12 months postoperatively and we found a correlation of SIM and global QoL score at 12 months postoperatively. Several patients of the “poor performer” (SIM < 40%) group reported high improvement of hearing-related QoL. Conclusions: Cochlear implantation provides a benefit in hearing-related QoL, even in some patients with low postoperative speech intelligibility results. Consequently, hearing-related QoL scores should be routinely used as outcome measure beside standard speech understanding tests, as well. Further studies with a prospective multi-centric design are needed to identify factors influencing post-implantation functional results and QoL in the patient group of long-term deafness.

## 1. Introduction

The standard outcome measure of cochlear implants (CIs) is the speech intelligibility test. However, quality of life (QoL) after cochlear implantation is as important as audiological performance. Several studies have evaluated the impact cochlear implantation on QoL and demonstrated significant postoperative improvements in hearing-related quality of life questionnaires [1,2,3,4,5], or in hearing-related domains of general health status questionnaires [2,5]. The Nijmegen Cochlear Implant Questionnaire (NCIQ) is a disease-specific questionnaire, which was adapted to different languages and is widely used to evaluate health-related QoL after cochlear implantation [2,6,7,8,9,10,11,12,13,14]. The NCIQ, developed 22 years ago, is still recommended as first-line measurement tool of QoL in patients after CI [15] and is used as a “legacy” patient-reported outcome measure for comparison of newer QoL measurement tools; for example, the Cochlear Implant Quality of Life (CIQOL)-35 Profile instrument and CIQOL-10 Global measure [16]. At the same time, the majority of NCIQ subdomains and the global QoL score have a poor construct validity in confirmatory factor analysis, only the basic sound performance and activity limitation show strong psychometric properties [16].

### 1.1. Hearing Outcome and QoL of Late Implanted Patients

A high proportion of late implanted (after >10 years deafness) patients has poor functional results [17], but little is known about the hearing-related QoL in this patient group. High user satisfaction rates have consistently been reported in studies on early-deafened late implanted CI users, even in subjects with almost negligible gain in auditory performance [17,18,19,20,21,22]. Early-deafened, late implanted patients can show significant postoperative improvements measured with the NCIQ [2,5,17,23] despite minimal gain in speech understanding. In 2011, van Dijkhuizen et al. only found a significant correlation between speech perception outcomes and the subdomain “advanced sound perception” of the NCIQ [4], whereas both Peasgood et al. [20] and Straatman et al. [5] found no significant correlations between auditory outcome measures and scores on the Glasgow Benefit Inventory. Additionally, Straatman et al. found no significant correlations between phoneme benefit scores and the generic Health Utilities Index 3, or the postoperative changes on the NCIQ [5]. The authors hypothesized that prelingually deafened adults, in contrast to postlingually deafened adults, might be satisfied with just minimal improvements in hearing abilities. The hearing outcome with CI relates significantly in early-deafened and late implanted patients with preoperative speech intelligibility [23], communication mode as a child [17,24,25,26], and preoperative speech-understanding scores [17,24,26,27]. However, no correlation was found with duration of deafness [17,23,24,27] or with etiology [25,27] in this patient group. Whereas post-lingually deafened patients generally reach their maximum performance between 6 to 12 months [28], early-deafened patients may continue improving their hearing performance over 1 year postoperatively [29]. However, other studies found no further improvement after 1 year of follow-up [23]. One may argue that poor performers have no benefit of cochlear implantation as their speech understanding remains under 40%. However, patients with early-onset hearing loss and poor speech discrimination may be able to understand suprasegmental cues after implantation [17]. Moreover, the integration of visual speech information with auditory information provided by the CI allows higher scores in audio-visual condition [30]. Consequently, the evaluation of the real gain of CI in prelingually deafened patients should enclose suprasegmental and audio-visual cues [23]. The factors determining the large inter-individual differences in speech intelligibility postoperatively seen in this patient group are mainly unknown, but a recent study identified the preoperative consonant-nucleus-consonant (CNC) word recognition score and the preoperative pure tone averages of the implanted ear as the factors explaining 63.5% of the variation in postoperative speech understanding with CI [31]. Indeed, significant study results can be found about the influence of other preimplantation factors, such as patients’ speech-understanding scores and preoperative hearing aid use [17,23,31].

### 1.2. The HiFocus Mid-Scala Electrode Array

The Cis HiRes 90K Advantage and the HiRes Ultra were commercially introduced 2012 and 2016, respectively. A new cochlear electrode array designed for the placement in the middle portion of the scala tympani, the HiFocus Mid-scala electrode (MSE) is the array used with these types of Cis since 2013 [32]. The MSE sits adjacent to the medial wall of the Scala tympani and is not in contact with the sensitive structures of the cochlea [33], minimizing the frictional forces on the lateral or medial wall during insertion [34]. The mean insertion force of MSE is less than 10 mN, corresponding to about 10% of the force expected for a normal straight electrode array with a similar insertion depth [34]. The MSE has a lower probability of dislocation into the scala vestibuli (scalar shift) than lateral or perimodiolar electrode arrays [35,36]. The functional advantages of the mid-scalar position are lower electrical thresholds, higher dynamic ranges and lower channel interaction compared to electrodes that are usually placed more peripherally in the Scala tympani [37]. The degree of hearing preservation is also higher with the MSE [38,39,40]. A comparison with the HiFocus 1J lateral wall electrode showed no difference in speech perception outcomes, despite the shallower insertion depth of MSE [41]. Additionally, MSE achieved a more consistent insertion depth than HiFocus 1J [39,41]. Battmer et al. compared the MSE with the Helix perimodiolar electrode array and they found that cochlear implantation with MSE led to significantly better postoperative SIM in quiet at each control interval from 3 to 12 months, at the same time, performance of both electrodes was in noise similar [42]. However, the biggest study to date with *n* = 328 participants comparing the outcome of MSE, lateral wall (LW) and perimodiolar (PM) electrodes showed no significant differences in hearing results [43]. However, studies dealing with quality of life (QoL) outcomes of MSE are lacking.

Taken together, the aim of this mono-centric retrospective cohort study was to evaluate the hearing performance and the QoL in a heterogenous patient group regarding duration and cause of deafness, but all implanted with the same CI electrode array (MSE). An emphasis was put on the performance and QoL of implantees with a long history (>10 years) of deafness, as controversial study results can be found on these subjects in the literature. The development of speech recognition and QoL scores should be analyzed over the follow-up period of one year and the relation of these two measures should be examined in this study, either.

## 2. Materials and methods

### 2.1. Patient Population

Epidemiological data are summarized in Table 1.

The cochlear implantations were carried out from December 2013 to November 2017 using the Advanced Bionics HiRes 90K Advantage (*n* = 27, 84.4%) or HiRes Ultra (*n* = 5, 13.6%) with the HiFocus Mid-scala electrode. The Naída Q70 or 90 sound processor was used in every patient.

### 2.2. Surgical Technique

All patients were operated by using a minimal retroauricular incision, extended antrotomy and regular posterior tympanotomy. All presented cases had an electrode insertion through the round window (RW) after intratympanic injection of dexamethasone and hyaluronic acid to the RW. Electrode insertions were carried out with consistent use of the insertion tool of AB after extended RW approach and with an insertion depth of 1 ¼ turns. Electrode tip fold over or kinking were ruled out by postoperative Stenvers view radiograph. The implantations have been performed by two operating surgeons (JEM, ON).

### 2.3. Postoperative Care

The first fitting of the cochlear implant was 2–4 weeks after surgery. All CI-recipients of this study went through an in-patient rehabilitation process in a specialized after-care center for cochlea implantees and received regular implant fittings at 3, 6, 9, 12, 18 and 24 months after activation of the CI. Depending on the hearing development of the patients, additional fittings were included, if necessary. After this period, yearly controls were carried out. Additionally, patients were motivated to train independently using CDs and Apps developed for auditory training.

### 2.4. Assessment of Audiological Performance

The audiometric protocol involved the following test battery: the Freiburger speech recognition test (monosyllables, numbers) was carried out routinely at each hearing measurement, preoperatively using headphones, with hearing aid and with CI postoperatively in free-field condition (signal on the implanted side, 45° azimuth, S_45_).

The HSM (Hochmair–Schulz–Moser) sentence-test with and without noise consists of three training lists and 30 test lists with each 20 daily life sentences in German. The measurements in noise were performed S_0_N_0_ at two levels (65 dB/65 dB or 65 dB/55 dB). The HSM sentence-test was introduced during the study and is not available for each participant of the study. The HSM sentence-test was utilized preoperatively, at 3 and 6 months postoperatively.

The OLSA (“Oldenburger Satztest”) or Oldenburger sentence-test with and without noise contains 40 test lists with 30 sentences (alternatively 25 test lists with each 20 sentences) uses an inventory of 50 words which can be randomly selected in a fixed order of noun/verb/number/adjective/grammatical object. The sentences are this way just partially meaningful; thus, not easy to retain and the test can be repeated. The OLSA measures the threshold at which test persons understand the 50% of the sentences in noise (S_0_N_-90_). However, if test persons do not achieve at least 80% speech understanding without noise, the measurements in noise are not rational. Additionally, patients having another native language as German have difficulty to understand the test sentences. Consequently, not all participants of the study could be tested preoperatively and at 12 months postoperatively.

The data of HSM und OLSA were not included in the analysis due to the incomplete dataset.

If masking of the better or normal hearing ear of the contralateral side was necessary, it was performed with a headphone and noise covering the speech frequencies.

Preoperative unaided speech reception thresholds (SRT_50_) and speech understanding for monosyllables (SIM) at 70 dB SPL using the Freiburger speech recognition test were compared with the postoperative aided thresholds at 1, 3, 6 and 12 months after cochlear implantation.

### 2.5. Measurements of the Quality of Life (QoL)

The Nijmegen Cochlear Implant Questionnaire (NCIQ) was used to assess the development of patients’ basic and advanced sound perception, speech production (so-called physical domains), self-esteem (psychological domain), activity limitations and social interactions (social domains) [2]. Each of the six subdomains of NCIQ contains ten items covering a total of 60 questions, prepared in a 5-point Liker scale. The first 55 questions can be answered with “never” (1), “sometimes” (2), regularly (3), “usually” (4) and “always” (5), and the last five items with “no” (1), “poor” (2), “moderate” (3), “good” (4) and “fairly good” (5). Participants can choose a sixth answer category “not applicable” in the case the item is not suitable. The total score for each subdomain is calculated as 1 = 0, 2 = 25, 3 = 50, 4 = 75 and 5 = 100, then the scores of each subdomain are summed and divided by the number of answers. The global score is the mean of the scores of the six subdomains. A higher score means a better QoL.

### 2.6. Statistical Analysis

Quantitative variables were expressed as means (±standard deviations). Data were summarized by descriptive statistics and normality was confirmed by the D′Agostino-Pearson test. Multiple comparisons were performed by one-way ANOVA (repeated measures, Tukey’s post-hoc test). Relationships between variables (QoL/SIM and deafness duration/SIM) were analyzed by correlation analysis (Pearson). Results were considered significant for *p* < *0*.05. All calculations were performed using Microsoft Excel (version 15.29, Microsoft Corporation, Redmond, WA, USA) and GraphPad Prism8 (GraphPad Software, San Diego, CA, USA).

## 3. Results

Individual epidemiological data, hearing results and quality of life scores are presented in Table 2.

### 3.1. Audiological Outcome

The functional results are presented in Table 2. The wearing time of the cochlear implant was less than 1 year in *n* = 2 (6.3%) ears, between 1 and 2 years in *n* = 22 (68.8%) ears and more than 2 years in *n* = 8 (25%) ears.

### 3.2. Freiburger Speech Recognition Test for Monosyllables (SIM) at 70 dB SPL

**Figure 1:**
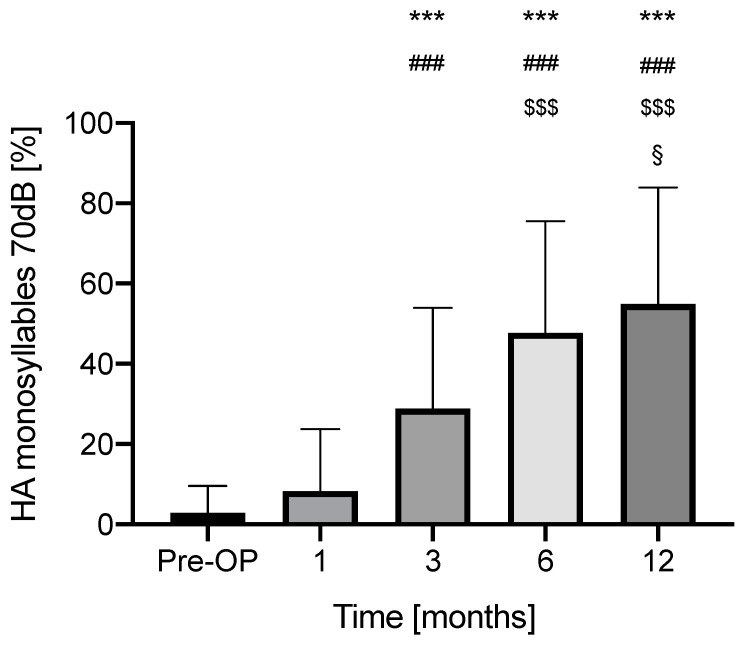
Aided (HA) speech understanding at 70 dB (Freiburger monosyllable test) preoperatively (with hearing aid), at 1, 3, 6 and 12 months postoperatively (with CI) in the whole patient cohort. Significant differences (*p* < 0.05) are indicated in comparison with * vs. Pre-OP, # vs. 1 month, $ vs. 3 months, § vs. 6 months. Significance levels are indicated as **/##/$$/§§ if *p* < 0.005 and as ***/###/$$$/§§§ if *p* < 0.0001.

The SIM values were as follows: preoperative (pre) 3.1 ± 7.0% (*n* = 32), at 1 month (1M) postoperatively 8.3 ± 15.43% (*n* = 32), at 3 months (3M) 29.7 ± 25.0%, at 6 months (6M) 48.5 ± 27.9% (*n* = 30) and at 12 months (12M) 54.8 ± 29.1% (*n* = 29). There was a significant and continuous improvement of SIM in Tukey’s multiple comparisons test, from 3 to 12 months with the use of the cochlear implant (pre vs. 3M: *p* < 0.0001, 95% CI −38.16 to 13.72; pre vs. 6M: *p* < 0.0001, 95% CI −58.71 to −31.10; pre vs. 12M: *p* < 0.0001, 95% CI −66.16 to −37.90; 1M vs. 3M: *p* < 0.0001, 95% CI −30.87 to −10.06; 1M vs. 6M: *p* < 0.0001, 95% CI −52.94 to −25.93; 1M vs. 12M: *p* < 0.0001, 95% CI −60.37 to −32.75; 3M vs. 6M: *p* = 0.0006, 95% CI −30.91 to −7.026; 3M vs. 12M: *p* < 0.0001, 95% CI −39.39 to −12.79; 6M vs. 12M: *p* = 0.0319, 95% CI −13.80 to −0.4468) (Figure 1). Only the preoperative SIM and the values at one month postoperatively did not significantly differ (pre vs. 1M: *p* = 0.1825, 95% CI −12.45 to 1.512).

For the group of patients with long-term (>10 years) deafness (*n* = 12, 42.9%) and for the cohort with short-term (<10 years) deafness (*n* = 16.57.1%) the following SIM values were measured: pre-OP 1 ± 3.2% and 4.4 ± 8.38% (*p* = 0.99), 1M 6.7 ± 15.4% and 8.9 ± 16.1% (*p* = 0.99), 3M 27.1 ± 30.7% and 30 ± 21.3% (*p* = 0.99), 6M 40.8 ± 32.1% and 51.8 ± 24.4% (*p* = 0.68), 12M 47.9 ± 33.6% and 57.1 ± 25.3% (*p* = 0.82), respectively. No significant differences were found between the groups.

For the group of patients having single-sided deafness (SSD *n* = 5, 17.9%) with normal hearing on the contralateral side and for the rest of the patients were the following SIM values measured: pre-OP 7.5 ± 15% and 2.5 ± 5.11% (*p* = 0.99), 1M 2 ± 4.5% and 9.2 ± 16.8% (*p* = 0.97), 3M 23 ± 19.2% and 30 ± 26.2% (*p* = 0.97), 6M 40 ± 22.9% and 48.8 ± 28.8% (*p* = 0.94), 12M 51 ± 27.9% and 54 ± 29.3% (*p* = 0.99), respectively. No significant differences were found between the groups.

Figure 1 Speech intelligibility for monosyllables.

### 3.3. Speech Reception Thresholds (SRT_50_)

**Figure 2:**
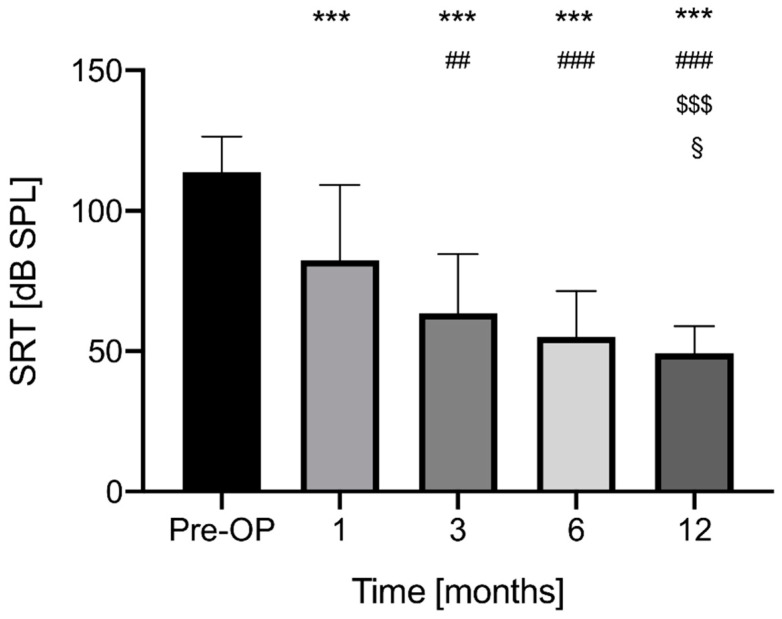
Speech reception thresholds (Freiburger speech test) preoperatively, at 1, 3, 6 and 12 months postoperatively in the whole patient cohort. Significant differences (*p* < 0.05) are indicated in comparison with * vs. Pre-OP, # vs. 1 month, $ vs. 3 months, § vs. 6 months. Significance levels are indicated as **/##/$$/§§ if *p* < 0.005 and as ***/###/$$$/§§§ if *p* < 0.0005.

If the SRT_50_ was not measurable due to patient´ deafness, the value was calculated with 120 dB. The following speech reception thresholds were measured: preoperative 113.6 ± 12.8 dB SPL (*n* = 32, measurable: *n* = 9, 28.1%), at 1 month postoperatively 82.4 ± 26.8 dB (*n* = 32, measurable: *n* = 22, 68.7%), at 3 months 63.5 ± 21.1 dB (*n* = 32, measurable: *n* = 30, 93.8%), at 6 months 55.1 ± 16.4 dB (*n* = 30), at 12 months 47.5 ± 7.8 dB (*n* = 29). (Figure 2). The SRT_50_ decreased significantly after 1 month postoperatively (pre vs. 1M: *p* < 0.0001, 95% CI 16.93 to 45.47), after 3 months (1M vs. 3M: *p* < 0.0001, 95% CI 6.212 to 31.60; pre vs. 3M: *p* < 0.0001, 95% CI 38.33 to 61.88) and up to 12 months (pre vs. 6M: *p* < 0.0001, 95% CI 47.89 to 69.17; pre vs. 12M: *p* < 0.0001, 95% CI 55.84 to 72.63; 3M vs. 6M: *p* = 0.1844, 95% CI −2.354 to 19.20; 3M vs. 12M: *p* = 0.0007, 95% CI 5.172 to 23.08; 6M to 12M: *p* = 0.1135, 95% CI −0.8647 to 12.27).

For the group of patients with long-term (>10 years) deafness and for the cohort with short-term (<10 years) deafness the following SRT_50_ values were measured: pre-OP not measurable and 94.3 ± 12.6 dB, 1M 64.2 ± 12.8 dB and 66.3 ± 7.4 dB (*p* = 0.97), 3M 60.7 ± 14.7 dB and 56.5 ± 7.6 dB (*p* = 0.75), 6M 57.0 ± 13.9 dB and 51.1 ± 8.8 dB (*p* = 0.46), 12M 52.6 ± 11 dB and 48.2 ± 7.7 dB (*p* = 0.72), respectively. No significant differences were found between the groups.

For the group of patients having single-sided deafness (SSD, *n* = 5, 17.9%) with normal hearing on the contralateral side and for the rest of the patients were the following SRT_50_ values were measured: pre-OP 102.5 ± 10.6 dB and 91.6 ± 12.8 dB (*p* = 0.18), 1M 68.5 ± 6.6 dB and 65.1 ± 9.1 dB (*p* = 0.97), 3M 58.4 ± 7.5 dB and 57.9 ± 11.3 dB (*p* = 0.99), 6M 53 ± 9.1 dB and 53.5 ± 11.9 dB (*p* = 0.99), 12M 49.4 ± 4.7 dB and 50 ± 9.9 dB (*p* = 0.99), respectively. No significant differences were found between the groups.

Figure 2 Speech reception thresholds.

There were *n* = 3 (10.1%) patients with a long history (>20 years) of deafness who had no speech intelligibility 12 months postoperatively. However, all three patients had SIM at the last control (patient 6: 45% SIM after 5 years, patient 7: 10% SIM after 4 years, and first implanted ear of patient 15: 40% SIM after 3 years of CI use, respectively). If these patients with long-time deafness excluded, SIM and SRT_50_ reached 12 months postoperatively 60.5 ± 24.1% and 47.5 ± 7.8 dB, respectively. No correlation between deafness time and SIM was found. Looking at the distribution of the whole cohort, *n* = 13 (40.6%) ears achieved a SIM of at least 70% after 12 months of CI wearing time. Five ears (15.6%) developed a SIM of 55–65%, another *n* = 5 (15.6%) ears 40–45%, respectively. Nine ears (28.1%) had a SIM of less than 40% (Table 2).

### 3.4. Measurements of Quality of Life

The hearing-related quality of life was registered with the Nijmegen Cochlear Implant Questionnaire (NCIQ). Preoperatively, *n* = 24 (85.7%) patients (*n* = 28, 87.5% of implanted ears) filled out the NCIQ. Nineteen patients (67.9%; *n* = 20 ears, 62.5%) and eighteen patients (64.3%; *n* = 20 ears, 71.4%) were eligible at the control at 6 and 12 months, respectively.

### 3.5. NCIQ Subdomain Scores

**Figure 3:**
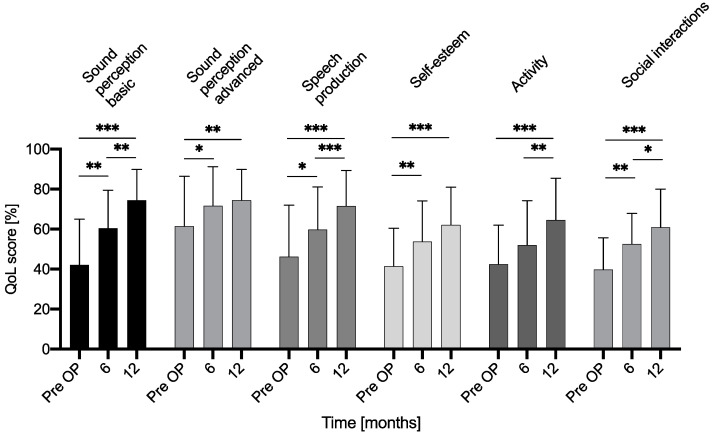
Results of Nijmegen Cochlear Implant Questionnaire before and 6 or 12 months after cochlear implantation in the whole patient cohort. Asterisks indicate a significant difference: * *p* < 0.05, ** *p* < 0.005, *** *p* < 0.0005.

In general, QoL subdomain scores significantly improved between the preoperative and postoperative period (Figure 3). We observe a progressive and significant improvement of QoL up to 12 months in the basic sound perception subdomain (pre vs. 6M: *p* = 0.0082, 95% CI −31.91 to −4.261; pre vs. 12M: *p* < 0.0001, 95% CI −45.40 to −19.27; 6M vs. 12M: *p* = 0.0011, 95% CI −22.08 to −6.057), in speech production (pre vs. 6M: *p* = 0.0307, 95% CI −26.02 to −1.176; pre vs. 12M: *p* < 0.0001, 95% CI −36.35 to −14.51; 6M vs. 12M: *p* < 0.0001, 95% CI −16.76 to −6.905) and in social interactions (pre vs. 6M: *p* = 0.0057, 95% CI −21.78 to −3.667; pre vs. 12M: *p* < 0.0001, 95% CI −29.62 to −12.72; 6M vs. 12M: *p* = 0.0410, 95% CI −16.55 to −0.3299).in The advanced sound perception reached its plateau at 6 months postoperatively (pre vs. 6M: *p* = 0.0094, 95% CI −18.00 to −2.444, pre vs. 12M: *p* = 0.0455, 95% CI −25.81 to −0.23336M vs. 12M: *p* = 0.7822, 95% CI −13.66 to 8.067), similar to the self-esteem (pre vs. 6M: *p* = 0.0055, 95% CI −20.91 to −3.557, pre vs. 12M: *p* < 0.0001, 95% CI −28.89 to −12.24, 6M vs. 12M: *p* = 0.0693, 95% CI −17.27 to 0.6097). The activity score showed a slow but significant increase up to 12 months postoperatively (pre vs. 6M: *p* = 0.0944, 95% CI −20.70 to 1.431, pre vs. 12M: *p* < 0.0001, 95% CI −33.06 to −11.37, 6M vs. 12M: *p* = 0.0097, 95% CI −22.06 to −3.107).

Figure 3 NCIQ QoL subdomain scores.

### 3.6. NCIQ Global Score

**Figure 4:**
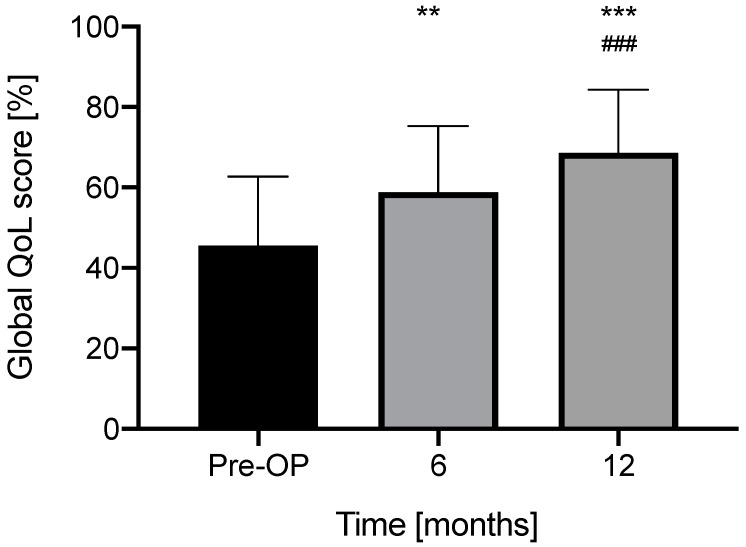
NCIQ global QoL scores preoperatively, and at 6 and 12 postoperative months. Asterisks indicate a significant difference in comparison with pre-OP: ** *p* < 0.005, *** *p* < 0.0005. Hashes indicate a significant difference in comparison with 6M: ### *p* < 0.0005 (for exact values see text).

The preoperative global QoL score (45.6 ± 17.2) increased progressively at both 6 months (58.6 ± 16.5, pre vs. 6M: *p* = 0.0014, 95% CI −21.32 to −5.241) and 12 months (68.6 ± 15.8, pre vs. 12M: *p* < 0.0001, 95% CI −30.73 to −15.33, 6M vs. 12M: *p* = 0.001, 95% CI −15.26 to −4.234) postoperatively in our patient group (Figure 4).

In the patient group with long-term deafness, out of 12 participants *n* = 9 (75%) filled out the NCIQ, in the patient cohort with short-term deafness out of 16 *n* = 14 (87.5%). The following global QoL scores were measured (long vs. short-term deafness): pre-OP 46.4 ± 20.4 and 45.1 ± 15.9 (*p* = 0.99), 6M 62.8 ± 20.6 and 55.1 ± 12.6 (*p* = 0.63), 12M 69.5 ± 17.8 and 67.8 ± 14.7 (*p* = 0.99), respectively. No significant differences were found between the groups.

In the patient group of SSD, out of five *n* = 4 participants (80%) filled out the NCIQ, in the rest of the patient cohort out of 23 participants *n* = 15 (65.2%). In the latter cohort, patients 5 and 15 filled out the NCIQ twice (sequential bilateral implantation). The following global QoL scores were measured (SSD vs. rest of the patient cohort): pre-OP 54.1 ± 18.2 and 44.0 ± 17 (*p* = 0.63), 6M 64.1 ± 14.4 and 56.8 ± 17 (*p* = 0.82), 12M 68.8 ± 15.6 and 68.5 ± 16.3 (*p* = 0.99), respectively. No significant differences were found between the groups.

Figure 4 NCIQ global QoL scores.

**Figure 5:**
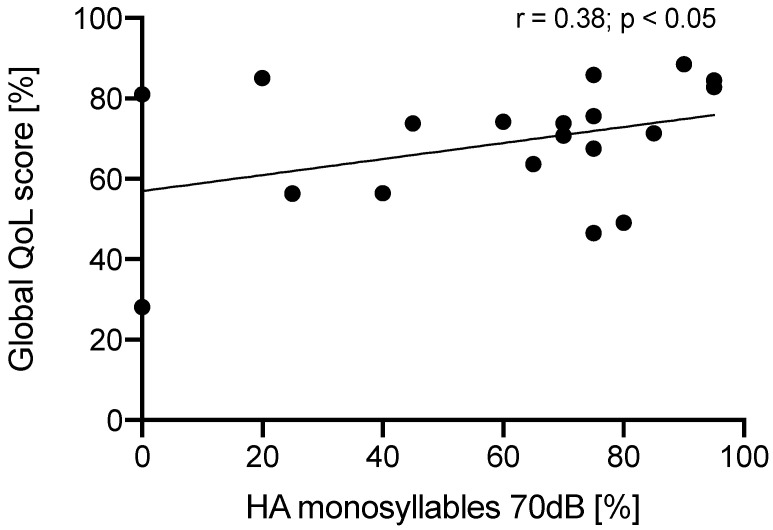
Correlation of global Quality of Life (QoL) scores of the Nijmegen Cochlear Implant Questionnaire with the CI-aided (HA) speech understanding at 70 dB SPL (Freiburger monosyllable test) postoperatively at 12 months.

The global QoL score correlated with the SIM of the whole patient cohort (Pearson correlation analysis, *r* = 0.3821, *p* = 0.0482) (Figure 5).

Figure 5 Correlation of SIM and NCIQ global QoL scores.

All data of this study are available as Supplementary Material (File S1, Excel-table).

## 4. Discussion

### 4.1. Summary of the Goals and Main Results of the Study

In this study, the development of speech recognition and QoL scores should be analyzed over time and the relation of these two measures should be examined. We found that in this patient cohort speech understanding for monosyllables (SIM) developed progressively up to one year after cochlear implantation. Some patients with a long history of deafness and with no speech recognition after one year follow-up developed a usable SIM after several years. All subdomain scores of the NCIQ increased significantly up to the end of the follow-up period of 12 months and the NCIQ global score increased significantly at both 6 and 12 postoperative months, either. We demonstrated a low correlation of SIM and global QoL score after 12 months CI wearing time in the whole patient cohort. Hearing-related QoL after cochlear implantation was in some cases high despite poor results of standard speech understanding tests.

### 4.2. Audiological Outcomes and Quality of Life after Cochlear Implantation

Eighteen (56.3%) ears in our study achieved a SIM of at least 50% after 12 months of CI wearing time. Nevertheless, nine ears (28.1%) underperformed having less than 40% SIM after 1 year of CI use. Looking closer at the cause of deafness and at the hearing of the contralateral side in this patient group, we can observe that three patients had a congenital or early-onset deafness and one patient had a removal of a vestibular schwannoma on the implanted side. These conditions are usually associated with a slower post-implantation hearing development and often result in a poor hearing performance after one year of CI use [17]. Interestingly, two patients of the congenital deafness group developed a usable speech understanding with the CI only after several years. High motivation and consequent CI use may have contributed to this hearing performance. Contrary to these cases, two patients in the underperforming group had single side deafness (patients 1 and 24, SIM at 12 months postoperatively 30 and 25%, respectively; Table 2). The latter patients may more rely on the contralateral side with a good hearing and therefore are less motivated to train with the CI, a possible reason for their poor speech understanding scores on the implanted ear. According to Muigg et. al, patients with SSD and long-term deafness are at higher risk to abandon the CI but if they do not discontinue CI use, they can have equal QoL benefit measured with NCIQ as implanted SSD patients with short-term (<10 years) deafness [44]. However, there is a bias in analyzing QoL results of patients with single-sided deafness as NCIQ measures QoL of both ears at the same time. Accordingly, patients with SSD having poor performance on the implanted ear and with normal contralateral hearing could have a good quality of life. In our study, those five patients with SSD (1, 8, 13, 20 and 24) were compared with the rest of the cohort and they had actually no difference regarding SIM at 12 months postoperatively (51 ± 27.9% vs. 54 ± 29.2% in the other 23 patients, *p* = 0.99) or global QoL score at 12 months (68.8 ± 15.6 vs. 68.5 ± 16.3, *p* = 0.99). We hypothesize that the masking of the normal hearing ear may be used during hearing training with the CI to achieve better audiological results on the implanted side, but we are not aware of a study that analyzed this theory.

The subgroup of early-deafened, late implanted patients in our cohort (*n* = 6) shows, in accordance with the literature, very heterogeneous audiological results postoperatively [17,29,31]. Three patients had usable (45 to 65%) SIM, the remaining three patients had no measurable benefit in terms of speech understanding after one year of CI wearing time. Nevertheless, two initial poor performer patients of this group achieved 40% and 45% SIM after 3 and 5 years, respectively.

Comparing the patient groups with long-term (>10 years) and short-term (<10 years) deafness, the latter showed by tendency a better SIM after 12 months CI wearing time (47.9 ± 33.6% vs. 57.1 ± 25.3%, *p* = 0.82), but the global QoL scores were after 1 year follow-up very similar (69.5 ± 17.8 vs. 67.9 ± 14.7). Accordingly, patients with a long-term deafness in our study had similar subjective benefit of the use of their cochlear implant as patients with short-term deafness.

With the use of CI, QoL scores increased significantly at the last measurement at 12 months in our whole patient group in all six subdomains of the NCIQ. The improvement of the global QoL score was in our study significant at both 6 and 12 months, either. Hirschfelder et al. published significant improvements in the global score and in all subdomain scores using NCIQ in 56 adult CI users [7]. The improvement of QoL concerns not only audiological aspects such as sound perception and speech production, but also psychosocial aspects such as self-esteem, activity level and social interaction. A significant improvement of QoL scores except for “activity” can be seen already after a wearing period of six months (Figure 3 and Figure 4). Furthermore, QoL scores at 12 months improved significantly in comparison with both preoperative and 6 months postoperative values, except for “sound perception advanced” and “self-esteem”, which reached their plateau after 6 months. Accordingly, the mean activity level and social interaction rose after implantation, which reinforces the thesis of postoperative re-socialization. In 2015, Mosnier reported in their study using the NCIQ a significant improvement of the QoL scores in all six subdomains after 6 months of CI use in elderly (65–85 years) patients. In contrast to our data, they measured stable results in QoL between 6 to 12 months after cochlear implantation [45]. Mean age at implantation of our cohort was 54.2 ± 15.9 years (range: 23–82) but the significant improvement of QoL scores in our study cannot be explained by implantees’ age alone. Notably, Plath et al. published recently their results of patients with a very similar age distribution (mean 55.3 ± 16.9 years, *n* = 100) at implantation and they reported no significant improvement of both global QoL score and NCIQ subdomain scores between 6 and 12 months postoperatively [15].

It would be interesting to determine the benefits of bilateral cochlear implantation compared to unilateral ones. Rader et al. reported that QoL measured with NCIQ increased by 23.7% after second CI in their patient cohort [46]. Unfortunately, there were only four bilaterally implanted patients in this study and only three of them filled out the NCIQ. Interestingly, the intrapersonal comparison demonstrated that two patients in our cohort who had been implanted sequentially showed gradual improvement of QoL scores after each cochlear implantation (patients 5 and 15, Table 2). This result is in line with the literature data and indicates the higher benefit of bilateral over unilateral cochlear implantation in patients with profound bilateral hearing loss or deafness [11,46].

Our data showed a low correlation of Freiburger SIM and global QoL score after one year CI wearing time (*r* = 0.384). Similarly, Hirschfelder et al. found a correlation of NCIQ total score, advanced sound perception, speech production subscore and speech recognition using the Freiburger SIM and HSM sentence-test [7]. Using the NCIQ and postoperative sentence or word recognition in quiet, Capretta found no broad correlation of hearing outcome and QoL in adult postlingually deafened CI users, significant correlations were only in speech related subscales of QoL measures [8]. A recent meta-analysis of QoL improvement after CI and associations with speech recognition abilities showed a very strong improvement of all hearing-related QoL measures, but the positive correlation was low between hearing-specific QoL and word recognition in quiet (*r* = 0.213), sentence recognition in quiet (*r* = 0.241), or sentence recognition in noise (*r* = 0.238) [47]. Apparently, traditional open-set speech tests may not fully capture the self-perceived benefit of a so-called poor performer CI-recipient. A good example in our cohort for this ambiguity is the patient 15 who had one year postoperatively 0% SIM on the first implanted ear but showed a considerable improvement of QoL scores which were near to (advanced sound perception) or higher than the mean scores of the subdomains in the cohort. In contrast, patient 7 with no measurable SIM 1 year postoperatively showed just minimal improvement of QoL scores. Our data appears to indicate that in early-deafened CI users, the benefit in terms of QoL scores is generally independent of auditory gains. Therefore, the assessment of subjective experiences after CI should be an integral component of the evaluation protocol, given that an individually experienced benefit is not fully captured by the auditory tests.

### 4.3. Strengths and Limitations of the Study

To our knowledge, our study is the first to demonstrate the audiological results together with the benefit on hearing-related QoL of the MSE. Furthermore, it is of advantage that data were collected with a specific CI to reduce the confounding effect of different CI systems using different algorithms, speech processors, or electrode arrays in performance analysis. The drawback of the study is the retrospective design, the incomplete QoL database and the low number of patients included. Further limitation of the study is the assessment of QOL which records the quality of life regarding both ears at the same time.

## 5. Conclusions

In our patient cohort, both hearing performance and hearing-related QoL scores improved progressively up to the last control at 12 months postoperatively. Early-deafened, late implanted patients may need several years for speech understanding development. Further studies with a prospective multi-centric design are needed to identify factors influencing post-implantation functional results and QoL in this special patient group. As self-perceived benefits of patients with CI can be high despite poor results of standard speech understanding tests, hearing-related QoL should also be routinely used as an outcome measure.

## Figures and Tables

**Table 1:** Summary of the epidemiological data.

Operated Patients	Operated Ears	Age at Implantation (Years)	Contralateral Ear	Cause of Deafness	Duration of Deafness (Years)
*n* = 28	*n* = 32	54.2 ± 15.85 (range: 23–82)	normal hearing *n* = 5 (15.6%) moderate hearing loss *n* = 5 (15.6%) severe hearing loss *n* = 4 (12.5%) profound hearing loss *n* = 16 (50.0%) congenital deafness *n* = 2 (6.3%) bilateral implantation *n* = 4 (12.5%)	SIHL *n* = 6 (18.8%) SSD *n* = 5 (15.6%) PSHL *n* = 11 (34.4%) congenital *n* = 4 (12.5%) genetic *n* = 1 (3.13%) surgery *n* = 1 (3.13%) surgical removal of vestibular schwannoma *n* = 1 (3.13%)	<10 years: *n* = 20 (62.5%) >10 years: *n* = 12 (37.5%)
Women *n* = 18 (64.3%)	left ears *n* = 15 (46.9%)				
Men *n* = 10 (35.7%)					
Bilaterally implanted *n* = 4 (14.3%)	right ears *n* = 17 (53.1%)				

**Table 2:** Epidemiological data, hearing results and quality of life scores of all patients in the study.

	Epidemiological Data				Hearing Performance Post-Op 12 Months (*n* = 32 Ears)		Quality of Life Pre-Op/Post-Op 12 Months (*n* = 20 Ears)						
Patient	Age	Contralateral Ear	Cause of Deafness	Duration of Deafness (Years)	SRT_50_ (dB)	SIM at 70 dB (%)	Sound Perception Basic	Sound Perception Advanced	Speech Production	Self-Esteem	Activity	Social Interactions	Global Score
1	34	normal hearing	SIHL, SSD	1	50	30							
2	81	moderate hearing loss	PSHL	1	47	80	25/58.3	50/50	45.8/62.5	55/43.8	25/30	25/50	37.6/49.1
3	59	profound hearing loss	congenital, Gusher	>10	57	45	22.5/77.5	47.5/75	20/62.5	37.5/70	57.5/85	55/72.5	40/73.8
4	50	profound hearing loss	congenital	>10	58	60							
5	64 (65)	first implanted ear, contralateral profound hearing loss	PSHL	1	43	75	17.5/63.9	30.6/36.1	20/50	30/37.5	8.3/50	22.2/41.7	21.4/46.5
		second implanted ear (bilateral cochlear implantation)			40	70	37.5/90	37.5/75	40/70	47.5/60	44.4/65.6	50/63.9	42.8/70.8
6	38	profound hearing loss	congenital	>10	63	0							
7	52	deafness	congenital	>10	67	0	15/32.5	30/32.5	2.5/15	27.5/45	41.7/22.5	35/21.4	25.3/28.2
8	59	normal hearing	PSHL, SSD	2	50	70	80.6/77.5	82.5/82.5	80/75	45/63.9	69.4/75	50/69.4	67.9/73.9
9	47	deafness	congenital	>10	43	65							
10	43	profound hearing loss	Genetic	4	42	85	47.5/72.5	95/100	50/72.5	44.4/66.7	37.5/50	41.7/66.7	52.7/71.4
11	63	moderate hearing loss	SIHL	>10	47	75	58.3/70	97.5/100	72.5/90	40/75	38.9/61.1	40.6/57.5	58/75.6
12	79	profound hearing loss	PSHL	2	43	65	41.7/78.1	62.5/80	16.7/62.5	42.5/52.5	32.1/63.9	27.8/45	37.2/63.7
13	44	normal hearing	SIHL, SSD	7	50	40	32.5/52.5	47.2/63.9	38.9/67.5	33.3/57.5	25/52.5	27.8/45	34.1/56.5
14	47	severe hearing loss	PSHL	1	55	45							
15	41 (42)	first implanted ear severe hearing loss	congenital	>10	72	0	52.5/75	52.5/75	62.5/82.5	72.5/90	75/88.9	62.5/75	62.9/81.1
		second implanted ear (bilateral cochlear implantation)	SIHL	1	39	95	58.3/72.5	80/80	57.5/87.5	80/92.5	67.5/87.5	72.5/87.5	69.3/84.6
16	28	simultaneous bilateral cochlear implantation profound hearing loss on both sides	Accident	4	58	45							
					62	35							
17	76	profound hearing loss	PSHL	1	32	95							
18	62	moderate hearing loss	SIHL	1	47	55							
19	46	bilaterally implanted within 1 week profound hearing loss on both sides	PSHL	>10	40	95	7.5/97.5	63.9/81.3	17.5/90	22.5/75	30.6/86.1	19.4/67.5	26.9/82.9
				1	32	95							
20	53	normal hearing	meningitis, SSD	1	42	90	80/85	92.5/91.7	80/87.5	62.5/80	55.6/90	55.6/97.2	71/88.6
21	23	moderate hearing loss	surgical removal of vestibular schwannoma	1	50	25							
22	49	profound hearing loss	PSHL	>10	55	25	60/72.5	63.9/75	60/66.7	27.5/35	57.5/44.4	46.8/44.4	52.6/56.3
23	65	profound hearing loss	PSHL	>10	40	75	15.6/82.5	33.3/82.5	18.8/77.8	8.3/45	0/56.3	21.9/61.1	16.3/67.5
24	68	normal hearing	SIHL, SSD	6	55	25	55/75	94.4/97.5	35.7/62.5	20/27.5	30/44.4	25/30.6	43.4/56.3
25	60	severe hearing loss	PSHL	>10	46	60	77.8/67.5	91.7/100	88.9/75	72.5/65	70/65	62.5/72.5	77.2/74.2
26	82	moderate hearing loss	Surgery	>10	43	75	57.5/92.5	82.1/90.6	70/87.5	55/87.5	58.3/91.7	27.8/65.6	58.5/85.9
27	78	severe hearing loss	PSHL	1	58	20	71.4/96.4	91.7/89.3	78.1/87.5	66.7/71.9	43.8/82.1	37.5/83.3	64.9/85.1
28	61	profound hearing loss	PSHL	1	53	40							
For the whole patient cohort:					47.5 ± 7.84	54.8 ± 29.09	42.1/74.46	61.4/77.89	46.2/71.60	41.5/62.06	42.4/64.60	39.7/60.89	45.6/68.58

Table 2 Patient-level data with epidemiologic characteristics, functional results and quality of life scores of Nijmegen Cochlear Implant Questionnaire. SIHL: sudden idiopathic hearing loss, SSD: single-sided deafness, PSHL: progressive sensorineural hearing loss.

## Data Availability

Data are contained within the article.

## Supplementary Materials

The following supporting information can be downloaded at: https://www.mdpi.com/article/10.3390/jcm11175156/s1, File S1: Dataset of the study title.

## References

[1] Schramm D., Fitzpatrick E., Séguin C. (2002). Cochlear implantation for adolescents and adults with prelinguistic deafness. Otol. Neurotol..

[2] Klop W.M., Boermans P.P., Ferrier M.B., van den Hout W.B., Stiggelbout A.M., Frijns J.H. (2008). Clinical relevance of quality of life outcome in cochlear implantation in postlingually deafened adults. Otol. Neurotol..

[3] Most T., Shrem H., Duvdevani I. (2010). Cochlear implantation in late-implanted adults with prelingual deafness. Am. J. Otolaryngol..

[4] Van Dijkhuizen J.N., Beers M., Boermans P.B.M., Briaire J.J., Frijns J.H.M. (2011). Speech intelligibility as a predictor of cochlear implant outcome in prelingually deafened adults. Ear Hear..

[5] Straatman L.V., Huinck W.J., Langereis M.C., Snik A.F.M., Mulder J.J. (2014). Cochlear implantation in late-implanted prelingually deafened adults: Changes in quality of life. Otol. Neurotol..

[6] Hinderink J.B., Krabbe P.F., Van Den Broek P. (2000). Development and application of a health-related quality-of-life instrument for adults with cochlear implants: The Nijmegen cochlear implant questionnaire. Otolaryngol.-Head Neck Surg..

[7] Hirschfelder A., Gräbel S., Olze H. (2008). The impact of cochlear implantation on quality of life: The role audiologic performance and variables. Otolaryngol. Head Neck Surg..

[8] Capretta N.R., Moberly A.C. (2016). Does quality of life depend on speech recognition performance for adult cochlear implant users?. Laryngoscope.

[9] Knopke S., Gräbel S., Förster-Ruhrmann U., Mazurek B., Szczepek A.J., Olze H. (2016). Impact of cochlear implantation on quality of life and mental comorbidity in patients aged 80 years. Laryngoscope.

[10] Olze H., Knopke S., Gräbel S., Szczepek A.J. (2016). Rapid positive influence of cochlear implantation on the quality of life in adults 70 years and older. Audiol. Neurotol..

[11] van Zon A., Smulders Y.E., Stegeman I., Ramakers G.G., Kraaijenga V.J., Koenraads S.P., Zanten G.A., Rinia A.B., Stokroos R.J., Free R.H. (2017). Stable benefits of bilateral over unilateral cochlear implantation after two years: A randomized controlled trial. Laryngoscope.

[12] Sladen D.P., Peterson A., Schmitt M., Olund A., Teece K., Dowling B., DeJong M., Breneman A., Beatty C.W., Carlson M.L. (2017). Hughes-Borst B, Driscoll CL Health-related quality of life outcomes following adult cochlear implantation: A prospective cohort study. Cochlear Implant. Int..

[13] Ketterer M.C., Knopke S., Häußler S.M., Hildenbrand T., Becker C., Gräbel S., Olze H. (2018). Asymmetric hearing loss and the benefit of cochlear implantation regarding speech perception, tinnitus burden and psychological comorbidities: A prospective follow-up study. Eur. Arch. Otorhinolaryngol..

[14] Häußler S.M., Knopke S., Wiltner P., Ketterer M., Gräbel S., Olze H. (2019). Long term benefit of unilateral cochlear implantation on quality of life and speech perception in bilaterally deafened patients. Otol. Neurotol..

[15] Plath M., Marienfeld T., Sand M., van de Weyer P.S., Praetorius M., Plinkert P.K., Baumann I., Zaoui K. (2022). Prospective study on health-related quality of life in patients before and after cochlear implantation. Eur. Arch. Otorhinolaryngol..

[16] McRackan T.R., Hand B.N., Velozo C.A., Dubno J.R. (2021). Cochlear Implant Quality of Life Consortium. Validity and reliability of the Cochlear Implant Quality of Life (CIQOL)-35 Profile and CIQOL-10 Global instruments in comparison to legacy instruments. Ear Hear..

[17] Caposecco A., Hickson L., Pedley K. (2012). Cochlear implant outcomes in adults and adolescents with early-onset hearing loss. Ear Hear..

[18] Hinderink J.B., Mens L.H., Brokx J.P., van den Broek P. (1995). Performance of prelingually and postlingually deaf patients using single-channel or multichannel cochlear implants. Laryngoscope.

[19] Zwolan T.A., Kileny P.R., Telian S.A. (1996). Self-report of cochlear implant use and satisfaction by prelingually deafened adults. Ear Hear..

[20] Peasgood A., Brookes N., Graham J. (2003). Performance and benefit as outcome measures following cochlear implantation in non-traditional adult candidates: A pilot study. Cochlear Implant. Int..

[21] Kaplan D.M., Shipp D.B., Chen J.M., Ng A.H.C., Nedzelski J.M. (2003). Early-deafened adult cochlear implant users: Assessment of outcomes. J. Otolaryngol..

[22] Bosco E., Nicastri M., Ballantyne D., Viccaro M., Ruoppolo G., Ionescu Maddalena A., Mancini P. (2013). Long-term results in late implanted adolescent and adult CI recipients. Eur. Arch. Otorhinolaryngol..

[23] van Dijkhuizen J.N., Boermans P.P., Briaire J.J., Frijns J.H. (2016). Intelligibility of the patient´s speech predicts the likelihood of cochlear implant success in prelingually deaf adults. Ear Hear..

[24] Yang W.S., Moon I.S., Kim H.N., Lee W.S., Lee S.E., Choi J.Y. (2011). Delayed cochlear implantation in adults with prelingual severe-to-profound hearing loss. Otol Neurotol..

[25] Zeitler D.M., Anwar A., Green J.E., Babb J.S., Friedmann D.R., Roland J.T., Waltzman S.B. (2012). Cochlear implantation in prelingually deafened adolescents. Arch. Pediatr. Adolesc. Med..

[26] Rousset A., Dowell R., Leigh J. (2016). Receptive language as a predictor of cochlear implant outcome for prelingually deaf adults. Int. J. Audiol..

[27] Kraaijenga V.J., Smit A.L., Stegeman I., Smilde J.J., van Zanten G.A., Grolman W. (2016). Factors that influence outcomes in cochlear implantation in adults, based on patient-related characteristics—A retrospective study. Clin. Otolaryngol..

[28] Lenarz M., Sönmez H., Joseph G., Büchner A., Lenarz T. (2012). Long-term performance of cochlear implants in postlingually deafened adults. Otolaryngol. Head Neck Surg..

[29] Santarelli R., De Filippi R., Genovese E., Arslan E. (2008). Cochlear implantation outcome in prelingually deafened young adults. A speech perception study. Audiol. Neuro-Otol..

[30] Craddock L., Cooper H., Riley A., Wright T. (2016). Cochlear implants for pre-lingually profoundly deaf adults. Cochlear Implant. Int..

[31] Debruyne J., Janssen M., Brokx J. (2017). Late cochlear implantation in early-deafened adults: A detailed analysis of auditory and self-perceived benefits. Audiol. Neuro-Otol..

[32] Global AB 2020 Reliability Report AB Technical Reports. Advanced Bionics. https://www.advancedbionics.com/content/dam/advancedbionics/Documents/Global/en_ce/Professional/Technical-Reports/Reliability/Global-AB-Reliability-Report-Autumn-2021.pdf.

[33] Gibson P., Boyd P. (2016). Optimal electrode design: Straight versus perimodiolar. Eur. Ann. Otorhinolaryngol. Head Neck Dis..

[34] Boyle P.J. (2016). The rational for a mid-scala electrode array. Eur. Ann. Otorhinolaryngol. Head Neck Dis..

[35] Zelener F., Majdani O., Roemer A., Lexow G.J., Giesemann A., Lenarz T., Warnecke A. (2020). Relation between scalar shift and insertion depth in human cochlear implantation. Otol. Neurotol..

[36] O’Connell B.P., Cakir A., Hunter J.B., Francis D.O., Noble J.H., Labadie R.F., Zuniga G., Dawant B.M., Rivas A., Wanna G.B. (2016). Electrode Location and Angular Insertion Depth Are Predictors of Audiologic Outcomes in Cochlear Implantation. Otol. Neurotol..

[37] Downing M. (2018). Electrode designs for protection of the delicate cochlear structures. J. Int. Adv. Otol..

[38] Kamali A., Gahm C., Palmgren B., Marklund L., Halle M., Hammarstedt-Nordenvall L. (2017). Use of a mid-scala and a lateral wall electrode in children: Insertion depth and hearing preservation. Acta Oto-Laryngol..

[39] Svrakic M., Roland J.T., McMenomey S.O., Svirsky M.A. (2016). Initial operative experience and short-term hearing preservation results with a mid-scala cochlear implant electrode array. Otol. Neurotol..

[40] Wanna G.B., O’Connell B.P., Francis D.O., Gifford R.H., Hunter J.B., Holder J.T., Bennett M.L., Rivas A., Labadie R.F., Haynes D.S. (2018). Predictive factors for short- and long-term hearing preservation in cochlear implantation with conventional-length electrodes. Laryngoscope.

[41] Van der Jagt M.A., Briaire J.J., Verbist B.M., Frijns J.H. (2016). Comparison of the HiFocus mid-scala and HiFocus 1J electrode array: Angular insertion depths and speech perception outcomes. Audiol. Neurotol..

[42] Battmer R.D., Scholz S., Gazibegovic D., Ernst A., Seidl R.O. (2020). Comparison of a Mid Scala and a Perimodiolar Electrode in Adults: Performance, Impedances, and Psychophysics. Otol. Neurotol..

[43] Fabie J.E., Keller R.G., Hatch J.L., Holcomb M.A., Camposeo E.L., Lambert P.R., Meyer T.A., McRackan T.R. (2018). Evaluation of Outcome Variability Associated with Lateral Wall, Mid-scalar, and Perimodiolar Electrode Arrays When Controlling for Preoperative Patient Characteristics. Otol. Neurotol..

[44] Muigg F., Bliem H.R., Kühn H., Seebacher J., Holzner B., Weichbold V.W. (2020). Cochlear implantation in adults with single-sided deafness: Generic and disease-specific long-term quality of life. Eur. Arch. Otorhinolaryngol..

[45] Mosnier I., Bebear J.P., Marx M., Fraysse B., Truy E., Lina-Granade G. (2015). Improvement of cognitive function after cochlear implantation in elderly patients. JAMA Otolaryngol. Head Neck Surg..

[46] Rader T., Haerterich M., Ernst B.P., Stöver T., Strieth S. (2018). Quality of life and vertigo after bilateral cochlear implantation: Questionnaires as tools for quality assurance. HNO.

[47] McRackan T.R., Bauschard M., Hatch J.L., Franko-Tobin E., Droghini H.R., Nguyen S.A., Dubno J.R. (2018). Meta-analysis of quality-of-life improvement after cochlear implantation and associations with speech recognition abilities. Laryngoscope.

